# Supplementary material for: Genetic Polymorphisms of MnSOD Modify the Impacts of Environmental Melamine on Oxidative Stress and Early Kidney Injury in Calcium Urolithiasis Patients
Source: Antioxidants (Basel). 2022 Jan 13;11(1):152. doi: 10.3390/antiox11010152 (PMC8773063; doi:10.3390/antiox11010152)
Supplement: Supplementary file 1 [file antioxidants-11-00152-s001.zip › antioxidants-1532915-supplementary.pdf]

Supplementary Materials

Genetic polymorphisms of *MnSOD* modify the impacts of environmental melamine on oxidative stress and early kidney injury in calcium urolithiasis patients

CC Liu, CF Wu, YC Lee, TY Huang, ST Huang, HS Wang, JH Jhan, SP Huang, CC Li, YS Juan, TJ Hsieh, YC Tsai, CC Chen, MT Wu

Table S1: STrengthening the REporting of Genetic Association studies (STREGA) reporting recommendations, extended from STROBE Statement

Item	Item no	STROBE Guideline	Extension for Genetic Association Studies (STREGA)	Checklist
**Title and Abstract**	1	(a) Indicate the study's design with a commonly used term in the title or the abstract.		Yes
		(b) Provide in the abstract an informative and balanced summary of what was done and what was found.		Yes
**Introduction**				
Background rationale	2	Explain the scientific background and rationale for the investigation being reported.		Yes
Objectives	3	State specific objectives, including any pre-specified hypotheses	***State if the study is the first report of a genetic association, a replication effort, or both.***	Yes
**Methods**				
Study design	4	Present key elements of study design early in the paper.		Yes
Setting	5	Describe the setting, locations and relevant dates, including periods of recruitment, exposure, follow-up and data collection.		Yes
Participants	6	(a) **Cohort study –** Give the eligibility criteria, and the sources and methods of selection of participants. Describe methods of follow-up.<br><br>**Case–control study –** Give the eligibility criteria, and the sources and methods of case ascertainment and control selection. Give the rationale for the choice of cases and controls.<br><br>**Cross-sectional study –** Give the eligibility criteria, and the sources and methods of selection of participants.	***Give information on the criteria and methods for selection of subsets of participants from a larger study, when relevant.***	Yes
		**(b) Cohort study** – For matched studies, give matching criteria and number of exposed and unexposed.<br><br>**Case–control study –** For matched studies, give matching criteria and the number of controls per case.		N/A
Variables	7	(a) Clearly define all outcomes, exposures, predictors, potential confounders, and effect modifiers. Give diagnostic criteria, if applicable.	***(b) Clearly define genetic exposures (genetic variants) using a widely –used nomenclature system. Identify variables likely to be associated with population stratification (confounding by ethnic origin).***	Yes
Data sources measurement	8*	(a) For each variable of interest, give sources of data and details of methods of assessment (measurement). Describe comparability of	***(b) Describe laboratory methods, including source and storage of DNA, genotyping methods and***	Yes

	9	assessment methods if there is more than one group.	***platforms (including the allele calling algorithm used, and its version), error rates and call rates. State the laboratory /centre where genotyping was done. Describe comparability of laboratory methods if there is more than one group. Specify whether genotypes were assigned using all of the data from the study simultaneously or in smaller batches.***	
Bias	9	(a) Describe any efforts to address potential sources of bias.	***(b) For quantitative outcome variables, specify if any investigation of potential bias resulting from pharmacotherapy was undertaken. If relevant, describe the nature and magnitude of the potential bias, and explain what approach was used to deal with this.***	Yes
Study size	10	Explain how the study size was arrived at.		N/A
Quantitative variables	11	Explain how quantitative variables were handled in the analyses. If applicable, describe which groupings were chosen, and why.	***If applicable, describe how effects of treatment were dealt with.***	Yes
Statistical methods	12	(a) Describe all statistical methods, including those used to control for confounding.	***State software version used and options (or settings) chosen.***	Yes
		(b) Describe any methods used to examine subgroups and interactions.		Yes
		(c) Explain how missing data were addressed.		Yes
		(d) **Cohort study –** If applicable, explain how loss to follow-up was addressed.<br><br>**Case–control study –** If applicable, explain how matching of cases and controls was addressed.<br><br>**Cross-sectional study –** If applicable, describe analytical methods taking account of sampling strategy.		Yes
		(e) Describe any sensitivity analyses.		Yes
			(f) State whether Hardy-Weinberg equilibrium was considered and, if so, how.	Yes
			(g) Describe any methods used for inferring genotypes or haplotypes.	Yes
			(h) Describe any methods used to assess or address population stratification.	Yes

			(i) Describe any methods used to address multiple comparisons or to control risk of false positive findings.	Yes
			(j) Describe any methods used to address and correct for relatedness among subjects.	Yes
**Results**				
Participants	13*	(a) Report the numbers of individuals at each stage of the study – e.g. numbers potentially eligible, examined for eligibility, confirmed eligible, included in the study, completing follow-up and analysed.	***Report numbers of individuals in whom genotyping was attempted and numbers of individuals in whom genotyping was successful.***	Yes
		(b) Give reasons for non-participation at each stage.		Yes
		(c) Consider use of a flow diagram.		N/A
Descriptive data	14*	(a) Give characteristics of study participants (e.g. demographic, clinical, social) and information on exposures and potential confounders.	***Consider giving information by genotype.***	Yes
		(b) Indicate the number of participants with missing data for each variable of interest.		Yes
		(c) **Cohort study –** Summarize follow-up time, e.g. average and total amount.		N/A
Outcome data	15*	**Cohort study –** Report numbers of outcome events or summary measures over time.	***Report outcomes (phenotypes) for each genotype category over time***	N/A
		**Case–control study –** Report numbers in each exposure category, or summary measures of exposure.	***Report numbers in each genotype category***	N/A
		**Cross-sectional study –** Report numbers of outcome events or summary measures.	***Report outcomes (phenotypes) for each genotype category***	Yes
Main results	16	(a) Give unadjusted estimates and, if applicable, confounder-adjusted estimates and their precision (e.g. 95% confidence intervals). Make clear which confounders were adjusted for and why they were included.		Yes
		(b) Report category boundaries when continuous variables were categorized.		Yes
		(c) If relevant, consider translating estimates of relative risk into absolute risk for a meaningful time period.		Yes
			(d) Report results of any adjustments for multiple comparisons.	Yes
Other analyses	17	(a) Report other analyses done – e.g. analyses of subgroups and interactions, and sensitivity analyses.		Yes
			(b) If numerous genetic exposures (genetic variants) were examined, summarize	Yes

		results from all analyses undertaken.	
		(c) If detailed results are available elsewhere, state how they can be accessed.	N/A
**Discussion**			
Key results	18	Summarize key results with reference to study objectives.	Yes
Limitations	19	Discuss limitations of the study, taking into account sources of potential bias or imprecision. Discuss both direction and magnitude of any potential bias.	Yes
Interpretation	20	Give a cautious overall interpretation of results considering objectives, limitations, multiplicity of analyses, results from similar studies, and other relevant evidence.	Yes
Generalizability	21	Discuss the generalizability (external validity) of the study results.	Yes
**Other information**			
Funding	22	Give the source of funding and the role of the funders for the present study and, if applicable, for the original study on which the present article is based.	Yes

STROBE: STtrengthening the Reporting of Observational Studies in Epidemiology

*Give information separately for cases and controls in case–control studies and, if applicable, for exposed and unexposed groups in cohort and cross-sectional studies.

Table S2 Relationships of SNPs of antioxidant enzyme genes with a urinary marker of oxidative stress, 8-OHdG, in 302 calcium urolithiasis patients.

		8-OHdG, N (%)			
MnSOD-rs4880		Low<50%	High≧50%	Crude OR (95% CI)	P value
Alleles	C	43 (14.2)	37 (12.3)	Ref	
	T	259 (85.8)	265 (87.7)	1.19 (0.74-1.91)	0.472
Genotypes	CC	4 (2.6)	4 (2.6)	Ref	
	CT	35 (23.2)	29 (19.2)	0.83(0.19-3.61)	0.802
	TT	112 (74.2)	118 (78.2)	1.05(0.26-4.32)	0.942
	P for trend				0.488
MnSOD-rs5746136					
Alleles	A	116 (38.4)	122 (40.4)	Ref	
	G	186 (61.6)	180 (59.6)	0.92 (0.66-1.28)	0.617
Genotypes	AA	22 (14.6)	26 (17.2)	1	
	AG	72 (47.7)	70 (46.4)	0.82 (0.43-1.59)	0.56
	GG	57 (37.7)	55 (36.4)	0.82 (0.41-1.61)	0.558
	P for trend				0.62
GPX1-rs1800668					
Alleles	T	13 (4.3)	8 (2.6)	Ref	
	C	289 (95.7)	294 (97.4)	1.65 (0.68-4.05)	0.271
Genotypes	TT+TC	13 (8.6)	7 (4.6)	Ref	
	CC	138 (91.4)	144 (95.4)	1.94 (0.75-5.00)	0.17
CAT-rs1001179					
Alleles	T	9 (3.0)	8 (2.6)	Ref	
	C	293 (97.0)	294 (97.4)	1.13 (0.43-2.97)	0.806
Genotypes	TT+TC	8 (5.3)	8 (5.3)	Ref	
	CC	143 (94.7)	143 (94.7)	1(0.37-2.74)	1
CAT-rs769217					
Alleles	T	144 (47.7)	145 (48.0)	Ref	
	C	158 (52.3)	157 (52.0)	0.99 (0.72-1.36)	0.935
Genotypes	TT	33 (21.9)	32 (21.2)	Ref	
	TC	78 (51.7)	81 (53.6)	1.07(0.60-1.91)	0.816
	CC	40 (26.4)	38 (25.2)	0.98(0.51-1.89)	0.951
	P for trend				0.933

Abbreviations: SNP=single-nucleotide polymorphism; 8-OHdG=8-oxo-2'-deoxyguanosine; N=number; *MnSOD*=manganese superoxide dismutase; *GPX1*=glutathione peroxidase; *CAT*= catalase

Table S3 Relationships of SNPs of antioxidant enzyme genes with a urinary marker of oxidative stress, MDA, in 302 calcium urolithiasis patients by using a new method of covariate-adjusted standardization plus creatinine adjustment.

		MDA, N (%)			
MnSOD-rs4880		Low<50%	High≧50%	Crude OR (95% CI)	P value
Alleles	*C*	48 (15.9)	32 (10.6)	Ref	
	T	254 (84.1)	270 (89.4)	1.59 (0.99-2.57)	0.056
Genotypes	*CC*	4 (2.6)	4 (2.6)	Ref	
	CT	40 (26.5)	24 (15.9)	0.60 (0.14-2.62)	0.50
	TT	107 (70.9)	123 (81.5)	1.15 (0.28-4.71)	0.85
	P for trend				0.067
MnSOD-rs5746136					
Alleles	*A*	125 (41.4)	113 (37.4)	Ref	
	G	177 (58.6)	189 (62.6)	1.18 (0.85-1.64)	0.318
Genotypes	*AA*	30 (19.9)	18 (11.9)	1	
	AG	65 (43.0)	77 (51.0)	1.97 (1.01-3.56)	0.047
	GG	56 (37.1)	56 (37.1)	1.68 (0.83-3.33)	0.148
	P for trend				0.322
GPX1-rs1800668					
Alleles	*T*	9 (3.0)	12 (4.0)	Ref	
	C	293 (97.0)	290 (96.0)	0.74 (0.31-1.79)	0.507
Genotypes	*TT+TC*	9 (6.0)	11 (7.3)	Ref	
	CC	142 (94.0)	140 (92.7)	0.81 (0.32-2.01)	0.644
CAT-rs1001179					
Alleles	*T*	9 (3.0)	10 (2.6)	Ref	
	C	293 (97.0)	294 (97.4)	1.13 (0.43-2.97)	0.806
Genotypes	*TT+TC*	8 (5.3)	8 (5.3)	Ref	
	CC	143 (94.7)	143 (94.7)	1.00 (0.37-2.74)	0.999
CAT-rs769217					
Alleles	*T*	142 (47.0)	147 (48.7)	Ref	
	C	160 (53.0)	155 (51.3)	0.94 (0.68-1.29)	0.684
Genotypes	*TT*	33 (21.9)	32 (21.2)	Ref	
	TC	76 (50.3)	83 (55.0)	1.13 (0.63-2.01)	0.687
	CC	42 (27.8)	36 (23.8)	0.88 (0.46-1.71)	0.714
	P for trend				0.675

Abbreviations: SNP=single nucleotide polymorphism; MDA=malondialdehyde; N=number; *MnSOD*=manganese superoxide dismutase; *GPX1*=glutathione peroxidase; *CAT*= catalase

Table S4. Relationships of SNPs of antioxidant enzyme genes with a urinary marker of oxidative stress, 8-OHdG, in 302 calcium urolithiasis patients by using a new method of covariate-adjusted standardization plus creatinine adjustment.

		8-OHdG, N (%)			
MnSOD-rs4880		Low<50%	High≧50%	Crude OR (95% CI)	P value
Alleles	C	41 (13.6)	39 (12.9)	Ref	
	T	261 (86.4)	263 (87.1)	1.06 (0.66-1.70)	0.810
Genotypes	CC	4 (2.6)	4 (2.6)	Ref	
	CT	33 (21.9)	31 (20.5)	0.94(0.22-4.09)	0.935
	TT	114 (75.5)	116 (76.9)	1.02(0.25-4.17)	0.981
	P for trend				0.817
MnSOD-rs5746136					
Alleles	A	118 (39.1)	120 (39.7)	Ref	
	G	184 (60.9)	182 (60.3)	0.97 (0.70-1.35)	0.868
Genotypes	AA	23 (15.2)	25 (16.6)	1	
	AG	72 (47.7)	70 (46.4)	0.89 (0.47-1.72)	0.738
	GG	56 (37.1)	56 (37.0)	0.92 (0.47-1.81)	0.809
	P for trend				0.869
GPX1-rs1800668					
Alleles	T	12 (4.0)	9 (3.0)	Ref	
	C	290 (96.0)	293 (97.0)	1.35 (0.56-3.25)	0.507
Genotypes	TT+TC	12 (7.9)	8 (5.3)	Ref	
	CC	139 (92.1)	143 (94.7)	1.54 (0.61-3.89)	0.358
CAT-rs1001179					
Alleles	T	10 (3.3)	7 (2.3)	Ref	
	C	292 (96.7)	295 (97.7)	1.44 (0.54-3.84)	0.463
Genotypes	TT+TC	9 (6.0)	7 (4.6)	Ref	
	CC	142 (94.0)	144 (95.4)	1.30 (0.47-3.60)	0.608
CAT-rs769217					
Alleles	T	139 (46.0)	150 (49.7)	Ref	
	C	163 (54.0)	152 (50.3)	0.86 (0.63-1.19)	0.370
Genotypes	TT	29 (19.2)	36 (23.8)	Ref	
	TC	81 (53.6)	78 (51.7)	1.47(0.82-2.62)	0.194
	CC	41 (27.2)	37 (24.5)	0.65(0.33-1.27)	0.206
	P for trend				0.155

Abbreviations: SNP=single-nucleotide polymorphism; 8-OHdG=8-oxo-2'-deoxyguanosine; N=number; *MnSOD*=manganese superoxide dismutase; *GPX1*=glutathione peroxidase; *CAT*= catalase

Table S5 Relationships of SNPs of antioxidant enzyme genes with a urinary marker of renal tubular injury, NAG, in 302 calcium urolithiasis patients by using a new method of covariate-adjusted standardization plus creatinine adjustment.

		NAG, N (%)			
MnSOD-rs4880		Low<50%	High≧50%	Crude OR (95% CI)	P value
Alleles	*C*	40 (13.2)	40 (13.2)	Ref	
	T	262 (86.8)	262 (86.8)	1.00 (0.63-1.60)	1
Genotypes	*CC*	5 (3.3)	3 (2.0)	Ref	
	CT	30 (19.9)	34 (22.5)	1.89 (0.42-8.58)	0.410
	TT	116 (76.8)	114 (75.5)	1.64 (0.38-7.01)	0.506
	P for trend				0.999
MnSOD-rs5746136					
Alleles	*A*	129 (42.7)	109 (36.1)	Ref	
	G	173 (57.3)	193 (63.9)	1.32 (0.95-1.85)	0.096
Genotypes	*AA*	29 (19.2)	19 (12.6)	Ref	
	AG	71 (47.0)	71 (47.0)	1.53 (0.79-2.97)	0.213
	GG	51 (33.8)	61 (40.4)	1.83 (0.92-3.63)	0.086
	P for trend				0.099
GPX1-rs1800668					
Alleles	*T*	11 (3.6)	10 (3.3)	Ref	
	C	291 (96.4)	292 (96.7)	1.10 (0.46-2.64)	0.824
Genotypes	*TT+TC*	11 (7.3)	9 (6.0)	Ref	
	CC	140 (92.7)	142 (94.0)	1.24 (0.50-3.08)	0.64
CAT-rs1001179					
Alleles	*T*	9 (3.0)	8 (2.6)	Ref	
	C	293 (97.0)	294 (97.4)	1.13 (0.43-2.97)	0.806
Genotypes	*TT+TC*	8 (5.3)	8 (5.3)	Ref	
	CC	143 (94.7)	143 (94.7)	1 (0.37-2.74)	1
CAT-rs769217					
Alleles	*T*	136 (45.0)	153 (50.7)	Ref	
	C	166 (55.0)	149 (49.3)	0.80(0.58-1.10)	0.166
Genotypes	*TT*	34 (22.5)	31 (20.5)	Ref	
	TC	68 (45.0)	91 (60.3)	1.47(0.52-2.62)	0.194
	CC	49 (32.5)	29 (19.2)	0.65(0.33-1.27)	0.206
	P for trend				0.155

Abbreviations: SNP=single-nucleotide polymorphism; NAG= N-acetyl b-D-glucosaminidase; N=number; *MnSOD*=manganese superoxide dismutase; *GPX1*=glutathione peroxidase; *CAT*= catalase

Table S6 Combined effects of *MnSOD* genetic polymorphism (rs4880) and urine melamine levels on the risk of a high urinary marker of oxidative stress, MDA, in 302 calcium urolithiasis patients by using a new method of covariate-adjusted standardization plus creatinine adjustment.

		MDA, N (%)				Model 1		Model 2	
rs4880 and Melamine	N	Low <50%	High ≧50%	Crude OR (95% CI)	P value	Adjusted OR (95% CI)	P value	Adjusted OR (95% CI)	P value
C allele+Melamine< 50%	48	31 (10.3)	17 (5.6)	1		1		1	
C allele+Melamine> 50%	32	17 (5.6)	15 (5.0)	1.61 (0.65-4.01)	0.307	1.43 (0.55-3.72)	0.461	1.37 (0.52-3.57)	0.523
T allele+Melamine< 50%	254	151 (50.0)	103 (34.1)	1.24 (0.65-2.37)	0.506	1.29 (0.65-2.52)	0.467	1.29 (0.66-2.54)	0.460
T allele+Melamine> 50%	270	103 (34.1)	167 (55.3)	2.96 (1.56-5.61)	0.001	2.69 (1.37-5.27)	0.004	2.61 (1.33-5.14)	0.006
P for trend					<0.001		<0.001		<0.001

		MDA, N (%)				Model 1		Model 2	
rs4880 and Melamine	N	Low <66.6%	High ≧66.6%	Crude OR (95% CI)	P value	Adjusted OR (95% CI)	P value	Adjusted OR (95% CI)	P value
T allele+Melamine< 50%	48	37 (9.2)	11 (5.5)	1		1		1	
T allele+Melamine> 50%	32	22 (5.4)	10 (5.0)	1.53 (0.56-4.18)	0.408	1.39 (0.49-3.97)	0.541	1.31 (0.45-3.77)	0.618
C allele+Melamine< 50%	254	193 (47.8)	61 (30.5)	1.06 (0.51-2.21)	0.870	1.06 (0.49-2.27)	0.883	1.06 (0.49-2.26)	0.887
C allele+Melamine> 50%	270	152 (37.6)	118 (59.0)	2.61 (1.28-5.34)	0.008	2.33 (1.11-4.91)	0.026	2.23 (1.05-4.71)	0.036
P for trend					<0.001		0.001		0.002

Abbreviations: *MnSOD*=manganese superoxide dismutase; MDA=malondialdehyde; N=number

Model 1: adjusting for age, sex, BMI, educational level, cigarette smoking, alcohol drinking, betel quid chewing, stone location, stone number, and stone size.

Model 2: adjusting for age, sex, BMI, educational level, cigarette smoking, alcohol drinking, betel quid chewing, stone location, stone number, and stone size, diabetes mellitus, hypertension, and dyslipidemia

Table S7 Combined effects of *MnSOD* genetic polymorphism (rs5746136) and urine melamine levels on the risk of a high urinary marker of renal tubular injury, NAG, in 302 calcium urolithiasis patients by using a new method of covariate-adjusted standardization plus creatinine adjustment.

		NAG, N (%)				Model 1		Model 2	
rs5746136 and Melamine	N	Low <50%	High ≧50%	Crude OR (95% CI)	P value	Adjusted OR (95% CI)	P value	Adjusted OR (95% CI)	P value
A allele+Melamine< 50%	112	64 (21.2)	48 (15.9)	1		1		1	
A allele+Melamine> 50%	126	65 (21.5)	61 (20.2)	1.25 (0.75-2.09)	0.391	1.19 (0.69-2.03)	0.533	1.16 (0.67-1.98)	0.601
G allele+Melamine< 50%	190	102 (33.8)	88 (29.1)	1.15 (0.72-1.84)	0.460	1.10 (0.67-1.79)	0.716	1.10 (0.67-1.80)	0.712
G allele+Melamine> 50%	176	71 (23.5)	105 (34.8)	1.97 (1.22-3.19)	0.006	1.92 (1.15-3.18)	0.012	1.87 (1.12-3.12)	0.017
P for trend					0.008		0.017		0.022

		NAG, N (%)				Model 1		Model 2	
rs5746136 and Melamine	N	Low <66.6%	High ≧66.6%	Crude OR (95% CI)	P value	Adjusted OR (95% CI)	P value	Adjusted OR (95% CI)	P value
A allele+Melamine< 50%	112	80 (19.9)	32 (15.8)	1		1		1	
A allele+Melamine> 50%	126	87 (21.6)	39 (19.3)	1.12 (0.64-1.96)	0.689	1.02 (0.87-1.82)	0.941	0.97 (0.54-1.74)	0.922
G allele+Melamine< 50%	190	132 (32.8)	58 (28.7)	1.10 (0.66-1.84)	0.720	1.02 (0.60-1.72)	0.953	1.03 (0.60-1.75)	0.924
G allele+Melamine> 50%	176	103 (25.7)	73 (36.2)	1.77 (1.07-2.95)	0.027	1.58 (0.94-2.72)	0.084	1.50 (0.87-2.56)	0.143
P for trend					0.024		0.071		0.106

Abbreviations: *MnSOD*=manganese superoxide dismutase; NAG= N-acetyl b-D-glucosaminidase; N=number;

Model 1: adjusting for age, sex, BMI, educational level, cigarette smoking, alcohol drinking, betel quid chewing, stone location, stone number, and stone size.

Model 2: adjusting for age, sex, BMI, educational level, cigarette smoking, alcohol drinking, betel quid chewing, stone location, stone number, and stone size, diabetes mellitus, hypertension, and dyslipidemia
